# HIV disease progression among women following seroconversion during a tenofovir-based HIV prevention trial

**DOI:** 10.1371/journal.pone.0178594

**Published:** 2017-06-28

**Authors:** Sharon A. Riddler, Marla Husnik, Gita Ramjee, Anamika Premrajh, Bomkazi Onini Tutshana, Arendevi Pather, Samantha Siva, Nitesha Jeenarain, Gonasagrie Nair, Pearl Selepe, Samuel Kabwigu, Thesla Palanee-Phillips, Ravindre Panchia, Felix Mhlanga, Lisa Levy, Edward Livant, Karen Patterson, Vanessa Elharrar, Jennifer Balkus

**Affiliations:** 1 University of Pittsburgh, Pittsburgh, PA, United States of America; 2 Statistical Center for HIV/AIDS Research & Prevention (SCHARP), Seattle, WA, United States of America; 3 HIV Prevention Research Unit, South African Medical Research Council, Westville, Kwa Zulu Natal, South Africa; 4 CAPRISA, University of KwaZulu Natal, Durban, South Africa; 5 The Aurum Institute, Klerksdorp, South Africa; 6 Makerere University-Johns Hopkins University Research Collaboration, Kampala, Uganda; 7 Wits Reproductive Health and HIV Institute, University of the Witwatersrand, Johannesburg, South Africa; 8 Perinatal HIV Research Unit, University of the Witwatersrand, Johannesburg, South Africa; 9 University of Zimbabwe, UZ-UCSF Collaborative Research Programme, Harare, Zimbabwe; 10 FHI 360, Durham, NC, United States of America; 11 Magee-Womens Research Institute, Pittsburgh, PA, United States of America; 12 Division of AIDS, NIH, Bethesda, MD, United States of America; 13 Fred Hutchinson Cancer Research Center, Seattle, WA, United States of America; 14 University of Washington, Seattle, WA, United States of America; Imperial College London, UNITED KINGDOM

## Abstract

**Background:**

Little is known regarding HIV disease outcomes among individuals who become infected with HIV while receiving antiretroviral medications for prevention. We compared HIV disease parameters among women who seroconverted while receiving tenofovir-containing oral or vaginal pre-exposure prophylaxis (PrEP) to placebo.

**Methods:**

Participants with HIV seroconversion in a randomized placebo-controlled trial of oral tenofovir, oral tenofovir/emtricitabine, and vaginal tenofovir gel (MTN-003) were followed in a longitudinal cohort study (MTN-015). The effect of oral and vaginal tenofovir-containing PrEP on HIV disease progression was compared to placebo using linear mixed effects and Cox proportional hazard models, as appropriate. Additional analyses were performed to compare the outcomes among participants with detectable tenofovir or emtricitabine in plasma at the first quarterly visit in MTN-003.

**Results:**

A total of 224 participants were included in the analysis; 93% from South Africa and 94% clade C virus. No differences in HIV RNA at steady state or the trajectory over 12 months were observed for each active arm compared to placebo; tenofovir gel recipients had higher CD4^+^ T cell counts (722 vs 596 cells/mm^3^; p = 0.02) at 90 days after estimated HIV seroconversion and higher average rates of change over 12 months compared to placebo (-181 vs -92 cells/mm^3^ per year; p = 0.08). With a median follow-up of 31 months, no significant differences were observed for time to CD4^+^ T cell count ≤350 cells/mm^3^, or the composite endpoint of CD4^+^ T cells ≤350 cells/mm^3^, initiation of antiretroviral therapy or death for each active arm compared to placebo. Additionally, there were no significant differences in the HIV RNA or CD4+ T cell counts at baseline, the change to month 12, or any disease progression outcomes among participants with oral drug detected and no oral drug detected compared to placebo.

**Conclusions:**

No clinically significant differences in HIV seroconversion outcomes were observed among women randomized to tenofovir-containing oral or vaginal PrEP regimens, however low overall adherence limits the generalizability of these findings.

## Introduction

Oral pre-exposure prophylaxis (PrEP) with tenofovir-emtricitabine for prevention of HIV has been recommended for high-risk individuals based on results of randomized, placebo-controlled, efficacy trials conducted among several populations.[1–8] Oral tenofovir-containing PrEP was not effective in two trials among heterosexual women in sub-Saharan Africa primarily due to poor adherence to the study intervention.[9, 10] The degree of oral PrEP effectiveness demonstrated in the trials with significant benefit ranges from 42 to 86% with a direct relationship between greater adherence and higher efficacy.[11] Additionally tenofovir formulated as a 1% vaginal gel was shown to be modestly effective in one randomized trial but was not effective in two other trials.[9, 12, 13]

Despite the overall success of oral tenofovir-containing PrEP, breakthrough infection with HIV occurs among PrEP users, especially with sub-optimal adherence. Additionally sporadic cases of infection despite high adherence have been recently reported in clinical practice.[14] The incidence of resistance to PrEP medications has been extensively reviewed. [15, 16] However, data are limited on other disease outcomes for individuals who acquire HIV infection while receiving PrEP.[1–5, 9, 10, 17] Investigations in animal models have demonstrated lower peak viremia and lack of CD4+ T cell depletion in acute infection in SHIV challenged macaques that acquired infection while on intermittent oral tenofovir PrEP, but the applicability of this model for understanding the outcomes of human infection with HIV with PrEP use may be limited.[18]

The MTN-003 trial assessed the safety and effectiveness of daily oral tenofovir disoproxil fumarate (TDF), oral TDF/emtricitabine (TDF/FTC) and 1% tenofovir (TFV) vaginal gel for HIV prevention among HIV uninfected heterosexual women in Sub-Saharan Africa.[9] All participants who acquired HIV in the MTN-003 trial were eligible for enrollment into an established, prospective, observational cohort study (MTN-015) designed to monitor participant outcomes following seroconversion in women who had been exposed to a biomedical HIV prevention strategy.[19] We assessed the impact of the tenofovir-based investigational study products on HIV-1 disease progression among the MTN-003 participants enrolled into MTN-015.

## Methods

### Study population

The study population for this analysis consisted of women who acquired HIV infection during participation in the MTN-003 (VOICE) trial and who enrolled into MTN-015. MTN-003 was conducted from September 2009 through August 2012 at 15 sites in South Africa, Uganda and Zimbabwe (ClinicalTrials.gov number NCT00705679).[9] MTN-003 participants were randomized equally to each of the five treatment arms: oral TDF, oral TDF/FTC, oral placebo, vaginal TFV gel, and vaginal placebo gel. Participants who were identified as HIV infected during MTN-003 were offered enrollment into MTN-015 unless the site investigator believed the participant was unable to provide consent or adhere to the study visits. Participants were educated and informed about the study prior to enrollment.

### Study design

MTN-015 was funded by the National Institutes of Health (ClinicalTrials.gov number NCT00514098). Written informed consent was obtained from each study participant. The protocol was approved initially and annually by an institutional review board or ethics committee at each study site and corresponding collaborating institutions in the US, if required (S1 Table).

Eligible participants were enrolled into MTN-015 at the time of or at any time after confirmation of HIV seroconversion in MTN-003. MTN-015 visits occurred at enrollment and at 1, 3, 6 months after first detection of HIV seroconversion, then every 6 months thereafter with a minimum duration of follow-up of 12 months. Participants who initiated antiretroviral medications for treatment (ART) or prevention of maternal to child transmission (PMTCT) switched to a revised visit schedule with study visits 2 weeks, 1, 3, and 6 months and every 6 months following ART/PMTCT initiation.

Clinical evaluations: Study visits included medical history, physical examination, behavioral questionnaires, risk reduction counseling, and laboratory testing. World Health Organization (WHO) criteria were used to classify AIDS-defining and HIV-related events.[20] Care provided at the sites included testing and treatment of sexually transmitted infections for participants and partners, HIV risk reduction counseling, family planning counseling, and provision of contraceptives. Participants were referred for HIV care and PMTCT either within the same site or at community ART clinics with an established referral relationship with the research site. Initiation of ART and PTMCT was based on local and/or WHO guidelines in place at the time of the study.[1]

In the MTN-003 study, HIV rapid testing kits were used at screening, enrollment, and monthly thereafter using the following assays: Determine HIV 1/2 (Abbott Diagnostic Division; Hoofddorp, Netherlands), OraQuick® (Orasure Technologies; Bethlehem, Pennsylvania, USA), and Uni-Gold Recombigen® HIV test (Trinity Biotech; Wicklow, Ireland). Positive rapid tests were confirmed by Western blot according to a standard algorithm.[9] Samples for plasma HIV-1 RNA and CD4^+^ T cell count were obtained at the seroconversion confirmation visit. Plasma TFV and FTC levels were measured by a validated ultra-performance liquid chromatographic-tandem mass spectrometric method in MTN-003 from plasma collected and stored at quarterly visits for all seroconverters on an active oral or gel arm as well as a subset of placebo recipients.[9, 21]

Upon enrollment into MTN-015, laboratory evaluations included CD4^+^ T cell count and HIV-1 RNA using validated methods approved by the MTN Network Laboratory at each visit. HIV-1 genotypic resistance testing was performed at the MTN Virology Core from samples obtained at the time of seroconversion in the MTN-003 trial and results were reported previously.[9] Additionally resistance testing could be requested by the site investigator for clinical indications.

### Statistical analysis

The primary objective of MTN-015 was to assess HIV disease progression 12 months post-seroconversion among participants who received an active product compared to placebo participants in the parent MTN study. MTN-003 included both an oral tablet placebo arm and a vaginal gel placebo arm; the placebo arms were combined into one comparison group for this analysis. The estimated seroconversion date was defined as the midpoint between the last negative and first positive HIV rapid test in MTN-003. Change in CD4^+^ T cell count and log_10_ plasma HIV RNA from ≥90 days following estimated seroconversion through 12 months after seroconversion were assessed using linear mixed effects models, where treatment arm, time (in years since HIV seroconversion) and an interaction term between the two were included as fixed effects and the intercept and time (change per year) were included as random effects with an unstructured covariance structure. Follow-up was censored at the date antiretrovirals were initiated (either for treatment [triple drug antiretroviral therapy] or for prevention of mother to child transmission of HIV). Descriptive statistics were used to summarize the proportion of participants who experienced an AIDS-defining illness at any time during follow-up. Kaplan–Meier curves and Cox proportional hazard models [unadjusted and stratified by country (South Africa versus Uganda and Zimbabwe)] were generated to assess time from the estimated HIV seroconversion to the following outcomes (at any point during follow-up): first CD4^+^ T cell count ≤ 200 cells/mm^3^, first CD4^+^ T cell count ≤ 350 cells/mm^3^, first AIDS-defining illness and initiation of ART. The same methods were used to assess a composite HIV disease progression endpoint, defined as the time to the first occurrence of CD4^+^ T cell count ≤ 350 cells/mm^3^, initiation of ART, or non-traumatic death. Participant follow-up was censored at the time of initiation of ART or PMTCT for all analyses, excluding those where ART initiation was the outcome. Because of low adherence to study product in MTN-003, we planned *a priori* to perform sensitivity analyses categorizing participants by whether or not study drug was detected in plasma at their first quarterly follow-up visit in MTN-003. Analyses were repeated comparing participants who had study drug detected compared to those who did not have study drug detected. In addition, as part of an exploratory analysis to better describe HIV disease progression by clade, we repeated the above analyses restricting the analysis population to those with clade C virus.

## Results

### Enrollment and characteristics of the study population

**Fig 1:**
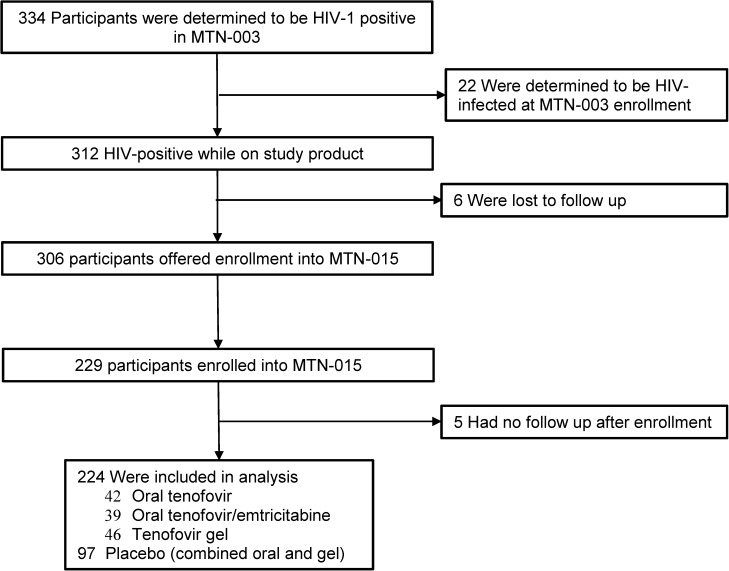
MTN-015 study population of participants from MTN-003 parent protocol.

A total of 334 women were identified to have HIV seroconversion during participation in MTN-003 (Fig 1), including 22 participants who were determined to have been acutely infected at the time of enrollment (based on the presence of HIV RNA in plasma).[9] Among the 312 women who had seroconversion while receiving study product in MTN-003, 229 (73%) were enrolled into MTN-015; 5 participants had no subsequent follow-up visits and were excluded from all analyses. The analysis population thus consists of 224 participants, 42 in the oral TDF arm, 39 in the oral TDF/FTC arm, 46 in the TFV gel arm and 97 in the combined placebo arms.

The baseline characteristics of evaluable participants at enrollment into MTN-015 by MTN-003 study arm are displayed in Table 1. The median age was 24 years, and the majority of the participants were enrolled in South Africa (93%) and were not married or living with a partner (84%). The infecting strain of HIV was clade C in 210 (94%) participants, with relatively few participants having clades A, CRF01_AE, and D (all of the non-clade C participants were enrolled in Uganda). The median time from the first positive HIV antibody test in MTN-003 to enrollment into MTN-015 was 2.1 months (Intraquartile range (IQR) 1.2, 3.5), and the median time from the estimated seroconversion date to enrollment into MTN-015 was 2.8 months (IQR 1.8, 4.3). Samples for HIV RNA and CD4^+^ T cell count were collected as part of the confirmation of seroconversion visit in MTN-003; therefore the first available results may be collected prior to enrollment in MTN-015. The median time from the first positive antibody test to the first HIV RNA was 30 days and for CD4 T-cell count was 30 days. The median initial CD4 T-cell count was 562 cells/mm^3^ and HIV RNA was 4.4 log_10_ copies/mL. The median follow-up time from estimated seroconversion date for the study participants was 31 months with a total of 548 person-years of observation. One death was reported during follow-up in a participant from the tenofovir gel arm. The cause of death was not identified but no AIDS-defining conditions were reported.

**Table 1:** Baseline characteristics of the study participants at enrollment into MTN-015.

	All (N = 224)	Oral TDF (N = 42)	Oral TDF/FTC (N = 39)	TFV gel (N = 46)	Placebo (N = 97)
Age (years)	**24**	24	23	24	23
	**(21, 26)**	(21, 27)	(22, 26)	(21, 27)	(21, 26)
South Africa	**208 (93%)**	41 (98%)	36 (92%)	42 (91%)	89 (92%)
Not married/living with partner	**189 (84%)**	39 (93%)	30 (77%)	36 (78%)	84 (87%)
Parity					
0	**31 (14%)**	7 (17%)	4 (10%)	8 (17%)	12 (12%)
1–2	**172 (77%)**	29 (69%)	31 (79%)	33 (72%)	79 (81%)
≥3	**21 (9%)**	6 (14%)	4 (10%)	5 (11%)	6 (6%)
Sexually Transmitted Infection					
*Chlamydia trachomatis*	**33/220 (15%)**	9/40 (23%)	4/39 (10%)	6/46 (13%)	14/95 (15%)
*Neisseria gonorrheae*	**20/221 (9%)**	2/40 (5%)	2/39 (5%)	8/46 (17%)	8/96 (8%)
Syphilis	**7/219 (3%)**	2/39 (5%)	0/39 (0%)	2/46 (4%)	3/95 (3%)
*Trichomonas vaginalis*	**17/205 (8%)**	1/39 (3%)	4/37(11%)	7/41 (17%)	5/88 (6%)
HIV Clade					
A	**3 (1%)**	0 (0%)	1 (3%)	1 (2%)	1 (1%)
C	**210 (94%)**	41 (98%)	35 (90%)	42 (91%)	92 (95%)
CRF01_AE	**5 (2%)**	1 (2%)	0 (0%)	2 (4%)	2 (2%)
D	**1 (0.5%)**	0 (0%)	0 (0%)	0 (0%)	1 (1%)
Missing	**5 (2%)**	0 (0%)	3 (8%)	1 (2%)	1 (1%)
HIV RNA* (log_10_ copies/ml)	**4.4**	4.5	4.4	4.1	4.4
	**(3.6, 5.0)**	(3.9, 5.1)	(3.9, 4.8)	(3.4, 4.7)	(3.6, 5.1)
CD4 T cell count*(cells/mm^3^)	**562**	478	665	609	523
	**(425, 731)**	(391, 673)	(484, 799)	(483, 783)	(409, 704)
Time from first + HIV rapid test to enrollment (months)	**2.1**	2.6	2.2	2.6	1.8
	**(1.2, 3.5)**	(1.3, 3.9)	(1.3, 3.4)	(1.5, 3.7)	(1.1, 3.1)
Time from estimated HIV SC to enrollment (months)	**2.8**	3.4	3.2	3.2	2.3
	**(1.8, 4.3)**	(1.8, 4.6)	(1.7, 4.2)	(2.2, 4.3)	(1.6, 4.1)
Follow up time from estimated HIV SC (months)	**30.7**	33.0	29.0	30.2	30.5
	**(24.4, 35.4)**	(23.7, 35.9)	(24.3, 35.3)	(19.2, 36.5)	(26.6, 35.2)
Total follow up time (person-years)	**548**	106	92	108	242

Median (IQR) unless noted

TDF: tenofovir disoproxil fumarate; FTC: emtricitabine; TFV: tenofovir; SC: Seroconversion

*First available result following estimated seroconversion (may be prior to enrollment). Median time from estimated seroconversion to the first available sample was 30 days for HIV RNA and 31 days for CD4 count.

### Clinical events and ART initiation

Twenty-one (9%) of the 224 participants reported an AIDS-defining event during follow-up. The most frequently reported event (14 participants) was unexplained severe weight loss (>10% of body weight). Seventy-four (33%) participants reported a total of 86 HIV-related events during follow-up. Unexplained moderate weight loss of <10% of body weight (62 participants; 68 events) was the most frequently reported HIV-related event.

Three (1%) participants initiated ART before enrollment into MTN-015 and were included in disease progression analyses based on follow-up time from their estimated seroconversion dates. All three participants met local CD4^+^ T cell treatment guidelines for ART initiation. In addition, 56 (25%) initiated ART after enrollment into MTN-015 and 13 (6%) received antiretroviral medications for PMTCT, including 3 who subsequently initiated combination ART. All initial ART regimens contained non-nucleoside reverse transcriptase inhibitor (NNRTI), and the most frequently reported initial ART combination was efavirenz, tenofovir and either lamivudine or emtricitabine (46/59 [78%]).

### HIV disease progression

#### Plasma HIV RNA and CD4 T-cell counts in the first year of follow-up

A total of 160 (71%) of participants were included in the month 12 HIV viral load analysis and 161 were included for CD4+ T cell count. Of the 64 participants excluded from this analysis, 17 (27%) had received antiretroviral mediations (ART or PMTCT) before the 12-month visit, 32 (50%) did not return for a visit within the 12-month visit window although they completed a later visit, 11 (17%) were lost-to-follow-up, and 4 (6%) participants had missing values for HIV RNA, one of whom had a CD4^+^ T cell count result. The overall mean change from baseline to month 12 for HIV RNA was -0.2 log_10_ (N = 160) and for CD4+ T cell count was -80 cells/mm^3^ (N = 161).

The results of the model comparing the first HIV RNA result (intercept) and HIV RNA trajectories (slope) to the 12-month visit are shown in Table 2. No significant difference among participants assigned to oral TDF, TDF/FTC or TFV gel compared to placebo was observed for the initial viral load or for the HIV RNA trajectory to the 12 month visit. The results of the model for CD4^+^ T cell count are also provided in Table 2. The first CD4^+^ T cell at 90 days after the estimated HIV seroconversion date was not significantly different among participants assigned to the oral TDF arm compared to placebo. However, CD4^+^ T cell counts were higher for the TFV gel arm (722 vs 596, p = 0.02) and there was a trend toward a higher CD4^+^ T cell count in the TDF/FTC arm (701 vs. 596, p = 0.07) at 90 days after the estimated HIV seroconversion date. CD4^+^ T cell counts decreased from baseline to month 12 for participants in all four groups and did not differ significantly among participants assigned to the oral TDF and TDF/FTC arms compared to placebo. However, the TFV gel arm trended toward a higher average rate of change over 12 months compared to placebo (-181 versus -92 cells/mm^3^ per year; p = 0.08).

**Table 2:** Linear mixed effects model comparing HIV-1 RNA (log_10_) and CD4 T-cell counts between the first result 90 days or more after the estimated HIV seroconversion date (intercept) and the 12-month visit (slope) by study arm.

MTN-003 Study Arm	Intercept (95% CI)		P-value	Slope (95% CI)		P-value
**Plasma HIV-1 RNA (Log**_**10**_ **copies/mL)**						
**Oral Tenofovir**	**4.35**	**(3.98, 4.73)**	**0.24**	**-0.27**	**(-0.66, 0.11)**	**0.31**
**Oral TDF/FTC**	**3.97**	**(3.58, 4.36)**	**0.63**	**0.30**	**(-0.07, 0.67)**	**0.15**
**Tenofovir gel**	**3.87**	**(3.52, 4.22)**	**0.33**	**0.24**	**(-0.09, 0.58)**	**0.19**
**Placebo**	**4.08**	**(3.83, 4.33)**	**—**	**-0.03**	**(-0.28, 0.22)**	**—**
**CD4 T-cell Count (cells/mm**^**3**^**)**						
**Oral Tenofovir**	**587**	**(495, 679)**	**0.87**	**-128**	**(-222, -34)**	**0.52**
**Oral TDF/FTC**	**701**	**(606, 795)**	**0.07**	**-138**	**(-227, -50)**	**0.39**
**Tenofovir gel**	**722**	**(637, 806)**	**0.02**	**-181**	**(-260, -102)**	**0.08**
**Placebo**	**596**	**(534, 657)**	**—**	**-92**	**(-152, -31)**	**—**

#### Time to ≤350 cells/mm^3^ and ART initiation

**Fig 2:**
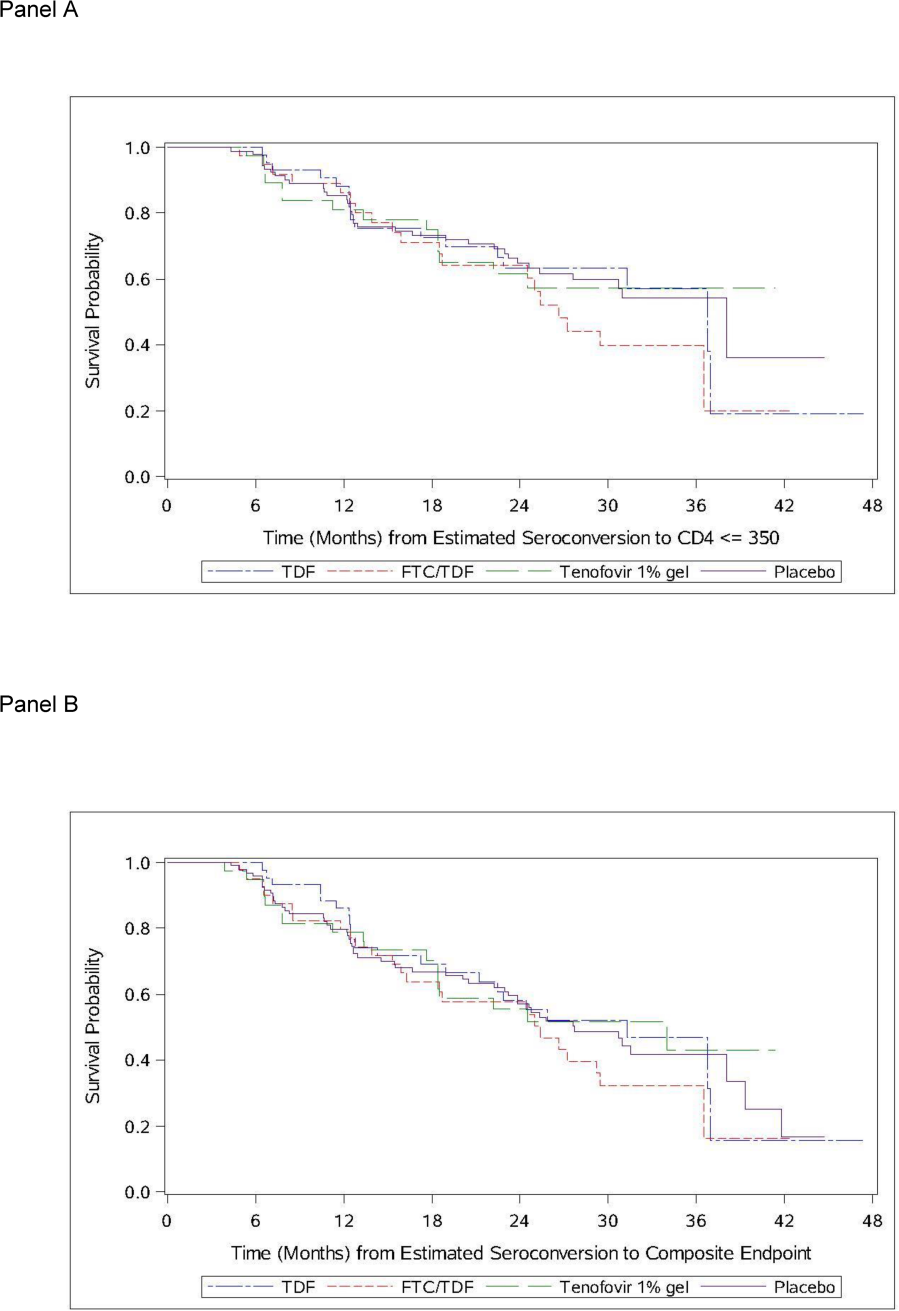
Time from HIV seroconversion to CD4+ T cell count ≤350 cells/mm^3^ (Panel A). Time from HIV seroconversion to the composite endpoint of CD4+ T cells ≤350 cells/mm^3^, initiation of ART or death (Panel B).

The results of unadjusted Cox proportional hazards model for the time to first CD4^+^ T cell count ≤350 cells/mm^3^ are shown in Table 3 and Fig 2. A total of 85 participants reached a CD4^+^ T cell count ≤350 cells/mm^3^ during follow-up. No statistically significant differences were observed for the time to first CD4^+^ T cell count ≤ 350 cells/mm^3^ among participants randomized to any active agent in MTN-003 compared to placebo. The time to initiation of ART, first AIDS-defining condition, and CD4^+^ T cell count ≤200 cells/mm^3^ also did not differ across the treatment arms (data not shown). For the composite HIV disease progression endpoint (first occurrence of CD4^+^ T cell count ≤350 cells/mm^3^, initiation of ART, or non-traumatic death), there were 92 events observed with no significant differences among participants randomized to an active agent in MTN-003 compared to placebo (Table 3). Similar results were seen for the models stratified by country as well as for the models limited to clade C virus only (data not shown).

**Table 3:** Unadjusted Cox proportional hazards model for time to first CD4 ≤ 350 and time to composite disease progression endpoint.

MTN-003 Study Arm	# of Events	Person-years	Incidence Rate^*^	Hazard Ratio (95% CI)		P-value
**Time to first CD4 T-cell count ≤350 cells/mm**^**3**^						
**Oral Tenofovir**	**19**	**65.8**	**28.9**	**1.41**	**(0.80, 2.46)**	**0.23**
**Oral TDF/FTC**	**14**	**66.4**	**21.1**	**1.00**	**(0.54, 1.86)**	**0.99**
**Tenofovir gel**	**17**	**81.1**	**21.0**	**1.01**	**(0.56, 1.80)**	**0.98**
**Placebo**	**35**	**168.1**	**20.8**	**1.00**	**—**	**—**
**Time to composite endpoint (CD4 ≤350 cells/mm**^**3**^**, initiation of ART, or non-traumatic death)**						
**Oral Tenofovir**	**24**	**67.8**	**35.4**	**1.24**	**(0.76, 2.02)**	**0.38**
**Oral TDF/FTC**	**18**	**67.1**	**26.8**	**0.94**	**(0.55, 1.61)**	**0.81**
**Tenofovir gel**	**22**	**82.9**	**26.5.**	**0.92**	**(0.56, 1.52)**	**0.74**
**Placebo**	**51**	**174.0**	**29.3**	**1.00**	**—**	**—**

*Per 100 person-years

#### Sensitivity analysis by drug detection at first quarterly visit in MTN-003

The results of the first quarterly assessment of drug detection from MTN-003 were used to define a subgroup of participants enrolled in MTN-015. The number of participants with detectable TFV and/or FTC in plasma was 10/37 (27%) for TDF, 13/38 (33%) for TDF/FTC, and 4/40 (10%) for TFV gel (5, 1, and 6 participants had missing results in the TDF, TDF/FTC, and TFV gel groups, respectively). The TFV gel group was excluded from further analysis as the number of participants with detectable TFV was low, and the TDF and TDF/FTC groups were combined. Thus the analysis population included 172 participants: 23 with drug detected and 52 with no drug detected from the combined oral arms, and 97 placebo.

A total of 119 (69%) of the 172 eligible participants had a viral load result available at the 12-month visit and prior to PMTCT or initiation of ART including 17/23 (74%) with drug detected, 34/52 (65%) with no drug detected, and 68/97 (70%) from the placebo arms; and 120/172 had CD4^+^ T cell results. In the linear mixed effects models, there were no significant differences in the HIV RNA or CD4^+^ T cell counts at baseline or the change to month 12 among participants with oral drug detected and no oral drug detected compared to placebo. There were no statistically significant differences among participants with and without oral drug detected compared to the placebo group for any of the disease progression outcomes. Results were similar after stratifying by country (data not shown).

## Discussion

In this analysis of a large cohort of women who acquired HIV infection while receiving oral or vaginal tenofovir-based PrEP, we observed no clinically significant impact of study treatment on subsequent HIV disease parameters, including viral set point and change in HIV RNA or CD4^+^ T cell count in the first year of infection. Post-estimated seroconversion CD4^+^ T cell counts for the tenofovir gel arm were higher relative to the placebo group, but similar mean CD4^+^ T cell counts were noted at 12 months. No differences were observed between MTN-003 study arms for HIV disease outcomes as measured by time to CD4 ≤ 350 cells/mm^3^, ART initiation, or death. This study comprises the largest cohort of seroconverters from a PrEP trial and includes participants who received oral and topical tenofovir-containing PrEP.

Several randomized clinical trials have demonstrated clear efficacy for the use of tenofovir for PrEP in men who have sex with men (MSM), heterosexual men and women, and intravenous drug users.[2–5] In these studies, participants with HIV seroconversion were monitored within the parent trial, generally for a minimum duration of 6 months. In the Pre-exposure Prophylaxis Initiative (iPrEx) study of TDF/FTC that enrolled HIV seronegative MSM and transgendered women, participants with seroconversion were followed with quarterly measurements of CD4^+^ T cell count and HIV RNA until completion of the trial.[4] No differences were observed between iPrEX participants with seroconversion from the TDF/FTC compared to the placebo group for CD4^+^ T cell count or HIV RNA at the time of seroconversion or subsequently for up to 60 weeks following seroconversion. However, the number of seroconverters who were followed and median duration of follow-up was not reported. Similarly, in the Bangkok Tenofovir Study that enrolled Thai men and women who inject drugs, HIV seroconverters were seen every four months.[3] In the Bangkok study, plasma HIV RNA was reported to be lower for the TDF arm compared to the placebo arm at the visit when seroconversion was first detected, but no differences were observed at later time points (month 4 to month 24) or in longitudinal analysis.

Three studies have reported more detailed outcomes of seroconverters in separate reports.[17, 22, 23] The Botswana TDF/FTC Oral HIV Prophylaxis Trial (TDF2 Study) compared 9 participants who seroconverted on TDF/FTC versus 24 participants who seroconverted in the placebo arm.[23] Among the 9 TDF/FTC participants, 4 were receiving study drug at the time of seroconversion and only 2 had detectable plasma levels. No significant differences in plasma HIV RNA values or CD4^+^ T cell counts were observed between the TDF/FTC and placebo recipients over a period of 120 weeks from seroconversion. Garrett, et al. reported the findings from extensive follow-up of 83 tenofovir gel and placebo participants who seroconverted while participating in the CAPRISA 004 trial.[12, 22] Although there were no differences in the median CD4^+^ T cell counts at quarterly intervals up to 24 months, there was a higher median HIV RNA among the tenofovir gel recipients (N = 32) compared to placebo. This difference was statistically significant only at the month 12 visit (TFV gel median HIV RNA approximately 0.5 log_10_ copies/ml higher than placebo; p = 0.016) but the interpretation is complicated by an unusually high proportion of participants with viral load below the limit of detection in the placebo arm. There was no difference in time to CD4^+^ T cell count <350 cells/mm^3^ between the tenofovir gel arm and placebo.

Finally, Grant et al. have reported follow up data on seroconverters from the FemPrEP trial of daily TDF/FTC comparing 33 seroconverters in the active arm to 35 who received placebo.[17] Tenofovir and emtricitabine concentrations were measured from all available samples prior to and at the time of seroconversion, and these values were used to estimate adherence (<1 tablet/month to >5 tablets/week). The authors reported no overall differences in CD4 and HIV RNA in the active and placebo arms but there was lower initial plasma HIV RNA among participants with evidence of higher adherence compared to those with lower or no drug detected, however this difference was not sustained at later time points.[17] Interestingly, investigators from the Partners PrEP study have recently reported a similar finding of lower initial HIV RNA and viral set point among HIV seroconverters with detectable plasma tenofovir levels compared to placebo.[24] Taken collectively, these two observations suggest that some impact of tenofovir on plasma viral load may be seen with higher adherence though the frequency, duration and clinical relevance of this occurrence are not yet well understood.

There are several potential reasons why we and others have observed little impact of tenofovir-containing PrEP on HIV disease progression. The most compelling reason is that in efficacy studies to date, only a minority of participants with seroconversion had detectable tenofovir at the time of seroconversion, and in many cases, adherence to the study product among those who seroconverted was poor throughout the trial period.[25] Thus there is minimal drug present with limited or no effect on the disease condition and rare development of drug resistance mutations.[15] Additionally, the currently approved medications for PrEP have relatively short half-life thus limiting exposure to the antiretroviral during periods of nonadherence. It will be critical to perform careful follow-up of participants who become infected during studies of investigational long acting depot formulations such as rilpivirine and cabotegravir due to the prolonged but sub-therapeutic plasma levels that are observed with these drugs after discontinuation of injections.[26, 27]

The findings of our study were limited by the low overall study product adherence in MTN-003.[9] When our analysis was restricted to the oral TDF/FTC participants with tenofovir detected at the first quarterly study visit, we again found no differences in disease progression; however, statistical power was limited, and unfortunately we did not have available samples for measurement of intracellular concentrations of tenofovir and emtricitabine, or for evaluation of drug concentrations at the time of first detection of seroconversion in most participants. Detailed analysis of the VOICE pharmacokinetic data has established that the majority of participants with detectable levels at the first quarterly time point will have detectable levels at subsequent visits.[28] Additionally, we were not able to assess for differences in HIV disease progression by HIV clade due to small numbers of participants infected with non-clade C virus; however, previous studies have demonstrated no differences in viral load by clade.[29]

In summary, we observed similar HIV disease outcomes in women who were receiving tenofovir-based oral or vaginal pre-exposure prophylaxis compared to placebo in the MTN-003 trial–similar to other studies these data are limited by the overall low adherence to the study products. Although reassuring, longitudinal follow-up of seroconverters from ongoing and future trials of antiretroviral drugs used for prevention are necessary to inform policy and best practices for PrEP.

## Supporting information

S1 Table Ethic committees and institutional review boards that provided study approval. (DOCX)
