# Supplementary material for: HIV disease progression among women following seroconversion during a tenofovir-based HIV prevention trial
Source: PLoS One. 2017 Jun 28;12(6):e0178594. doi: 10.1371/journal.pone.0178594 (PMC5489164; doi:10.1371/journal.pone.0178594)
Supplement: S1 Table — (DOCX) [file pone.0178594.s001.docx]

| **Name and Location of Implementing Site** | **Ethics Committees/IRBs that provided approval** |
| --- | --- |
|  |  |
| Medical Research Council Sites:  Chatsworth  Botha’s Hill  Isipingo  Overport  Tongaat  Verulam  Umkomaas  (Durban, South Africa) | **UKN BREC: University of KwaZulu-Natal Biomedical Research Ethics Committee** |
| CAPRISA Sites:  eThekwini  (Durban, South Africa)  Aurum  (Klerksdorp, South Africa) | **UKN BREC: University of KwaZulu-Natal Biomedical Research Ethics Committee** |
| UZ-UCSF HIV Prevention Trials Unit Sites:  Seke South  Spilhaus  Zengeza  (Harare, Zimbabwe) | **MRCZ: Medical Research Council of Zimbabwe**  **RCZ: Research Council of Zimbabwe**  **UCSF-CHR: University of California, San Francisco- Human Research Protection Program & IRB (formerly CHR)**  **UZ JREC: University of Zimbabwe Joint Research Ethics Committee** |
| Wits RHI CRS (Johannesburg, South Africa)  Perinatal HIV Research Unit  (Soweto, South Africa) | **Wits HREC: Wits University Human Research Ethics Committee** |
| MU-JHU Research Collaboration  (Kampala, Uganda) | **Johns Hopkins University IRB**  **NARC: National HIV/AIDS Research Committee**  **UNCST: Uganda National Council for Science and Technology** |
